# Suboptimal blood pressure control and its associated factors among people living with diabetes mellitus in sub-Saharan Africa: a systematic review and meta-analysis

**DOI:** 10.1186/s13643-022-02090-4

**Published:** 2022-10-15

**Authors:** Yonas Akalu, Yigizie Yeshaw, Getayeneh Antehunegn Tesema, Sofonyas Abebaw Tiruneh, Achamyeleh Birhanu Teshale, Dessie Abebaw Angaw, Misganew Gebrie, Baye Dagnew

**Affiliations:** 1 Department of Physiology, School of Medicine, College of Medicine and Health Sciences, University of Gondar, P. O. Box 196, Gondar, Ethiopia; 2 Department of Epidemiology and Biostatistics, Institute of Public Health, College of Medicine and Health Sciences, University of Gondar, P. O. Box 196, Gondar, Ethiopia; 3 Department of Public Health, College of Health Sciences, Debre Tabor University, Debre Tabor, Ethiopia; 4 Department of Human Anatomy, School of Medicine, College of Medicine and Health Sciences, University of Gondar, P. O. Box 196, Gondar, Ethiopia

**Keywords:** Suboptimal blood pressure control, Hypertension, Diabetes mellitus, Sub-Saharan Africa, Meta-analysis

## Abstract

**Background:**

Suboptimal blood pressure control among people living with diabetes mellitus (DM) is one of the primary causes of cardiovascular complications and death in sub-Saharan Africa (SSA). However, there is a paucity of evidence on the prevalence and associated factors of suboptimal blood pressure control in SSA. Therefore, this review aimed to estimate its pooled prevalence and associated factors among people living with DM in SSA.

**Methods:**

We systematically searched PubMed, African Journals OnLine, HINARI, ScienceDirect, Google Scholar, and direct Google to access observational studies conducted in SSA. Microsoft Excel spreadsheet was used to extract the data, which was exported into STATA/MP version 16.0 for further analyses. Heterogeneity across studies was checked using Cochran’s Q test statistics and *I*^2^ test, and small study effect was checked using Funnel plot symmetry and Egger’s statistical test at a 5% significant level. A random-effects model was used to estimate the pooled prevalence and associated factors of suboptimal blood pressure control at a 95% confidence interval (CI) and significance level of *p* < 0.05.

**Results:**

Of the 7329 articles retrieved, 21 articles were eligible for the meta-analysis. After performing random-effects model, the pooled prevalence of suboptimal blood pressure control was 69.8% (95% *CI*: 63.43, 76.25%). Poor adherence to antihypertensive treatment (*OR* = 1.7; 95% *CI*: 1.03–2.80, *I*^2^ = 0.0%, *p* = 0.531) and overweight (*OR* = 2.4, 95% *CI*: 1.57–3.68, *I*^2^ = 0.00%, *p* = 0.47) were significantly associated with suboptimal blood pressure control.

**Conclusions:**

The prevalence of suboptimal blood pressure control among diabetic patients in SSA was high, and poor adherence to antihypertensive treatment and overweight were significantly associated with suboptimal blood pressure control. Hence, there is an urgent need for initiatives to improve and control hypertension, and preventive measures should concentrate on modifiable risk factors.

**Systematic review registration:**

PROSPERO CRD42020187901.

**Supplementary Information:**

The online version contains supplementary material available at 10.1186/s13643-022-02090-4.

## Background

Hypertension is the worldwide leading cause of cardiovascular diseases (CVD) and deaths [1] and accounts for around 7.5 million yearly deaths [2]. Globally, an estimated 1.13 billion people are hypertensive, most (two-thirds) living in low- and middle-income countries [3]. The highest prevalence of hypertension in the world is observed in SSA [4, 5]. It tends to occur more commonly with diabetes, and as many as 70 to 80% of diabetic patients suffer from hypertension, which worsens and accelerates the progression of both micro and macrovascular complications of diabetes and results in a 7.2-fold increase in the risk of mortality [6–9].

Therefore, it is imperative to control CVD risk and mortality in diabetes patients, and the most effective and powerful intervention to reduce it is controlling blood pressure by integrated use of lifestyle modifications and appropriate regimen and dose of antihypertensive medications [10, 11]. The benefits of tight BP control in patients with diabetes exceed the benefits of tight glycemic control and extend to the prevention of macrovascular and microvascular complications [12, 13]. Many randomized controlled trials and the United Kingdom Prospective Diabetes Study (UKPDS) showed that strict BP control in patients with hypertension and diabetes reduces the risk of stroke, coronary heart disease, congestive heart failure, macrovascular and microvascular complications, and death [12, 14, 15].

A meta-analysis has revealed that a 10-mm Hg reduction in systolic blood pressure reduced the risk of major cardiovascular disease events by 20%, coronary heart disease by 17%, stroke by 27%, heart failure by 28%, and all-cause mortality by 13% [16]. Lowering blood pressure to treatment targets is, therefore, a priority in individuals with diabetes to prevent complications [17–19]. Even though different guidelines differ in their recommendations on BP targets in diabetic patients [20], many guideline committees had recommended that in patients with DM and hypertension, the target systolic and diastolic BP should be below 130 and 80 mm Hg, respectively [6, 20, 21]. However, most hypertensive diabetic patients fail to meet the recommended BP target. A study in the USA by Andros et al. reported that suboptimal blood pressure control among individuals with diabetes was still high and remains a major public health concern causing economic burden [22]. In a longitudinal cohort study of 30,228 diabetic patients, only 43 and 30% of European American and African American diabetic hypertensive patients, respectively, demonstrated a target blood pressure of 130/80 mmHg [23]. In SSA, rates of BP control range between 11 and 35% [24, 25], and the cardiovascular complications in this region diabetic individuals are attributed to the suboptimal blood pressure control [26]. A study carried out in six specialized diabetes care centers of six SSA showed an overall suboptimal BP control in T2DM individuals despite adherence to guidelines [25]. This implies the presence of other factors attributed to this suboptimal BP control, including demographic, health literacy, and socioeconomic characteristics [27]. Moreover, being overweight and noncompliance with antihypertensive drugs were strongly associated with uncontrolled hypertension [28, 29].

Even though extensive efforts to develop interventional BP control strategies to decrease the risk of complications have been made in the past several decades, there is still a significant rise in the risk of complications in diabetic patients with hypertension, and the control of BP is suboptimal. To solve this problem and meet the blood pressure target, understanding the pooled prevalence and risk factors of blood pressure control particularly in SSA is important. Therefore, this systematic review and meta-analysis aimed to determine the pooled prevalence and associated factors of suboptimal blood pressure control among people living with DM in SSA.

By pooling the findings of 21 studies, this study determined the pooled prevalence and associated factors of suboptimal blood pressure control among DM patients which in turn may help clinicians and policymakers to design effective intervention strategies to improve blood pressure control among the diabetic population in the most affected region, SSA. Moreover, the result of this study will be a baseline for future studies that possibly determine other possible causes of the high prevalence of suboptimal blood pressure control.

### Review questions

1. What is the estimated pooled prevalence of suboptimal blood pressure control among diabetes mellitus patients in sub-Saharan African countries?
2. What are the associated factors of suboptimal blood pressure control among diabetes mellitus patients in sub-Saharan African countries?

## Methods

The protocol for this review has been registered in PROSPERO with a protocol number CRD42020187901, URL: https://www.crd.york.ac.uk/PROSPERO/#myprospero. This systematic review and meta-analysis followed the Preferred Reporting Items for Systematic Reviews and Meta-Analysis guidelines (S1 Table, PRISMA Checklist) [30].

### Eligibility criteria

#### Inclusion criteria

- Setting/context: This review included all studies conducted in SSA countries. All included studies were published.
- Population and condition: The review included studies involving people living with type 1 or type 2 diabetes mellitus.
- Study design: All observational (cross-sectional) studies that have reported the prevalence and/or associated factors of suboptimal blood pressure control among people living with type 1 or type 2 diabetes mellitus were included.
- Language: Studies written in the English language were included.
- Outcome: The outcome variable of this study was suboptimal blood pressure. In the primary studies, the outcome variable was defined using different blood pressure cut points. Included studies defined suboptimal blood pressure control in diabetes as follows: (1) systolic blood pressure (SBP) ≥ 140 mmHg and/or a diastolic blood pressure (DBP) ≥ 90 mmHg, (2) *SBP* ≥ 140 mmHg and/or a *DBP* ≥ 80 mmHg, and (3) *SBP* ≥ 130 mmHg and/or DBP ≥ 80. The latest BP cut point to define suboptimal blood pressure control among DM patients is SBP ≥ 130 mmHg and/or DBP ≥ 80 [31–33].
- Publication year: Studies published before July 20, 2020, were included.

#### Exclusion criteria

Studies that did not report the prevalence of suboptimal blood pressure control among type 1 or type 2 diabetes patients, case reports, case series, letters to the editors, and studies conducted on specific populations were excluded.

### Search strategies

PubMed, African Journals OnLine, HINARI, ScienceDirect, Google Scholar, and direct Google search were used to access relevant studies for this review. Moreover, reference lists of eligible studies were retrieved to account for the missed studies in the database searching. All studies reporting the proportion or prevalence of suboptimal blood pressure control among people living with DM (either T1DM or T2DM) in SSA countries were the target of this review.

A search strategy was established for each database by combining MeSH (Medical Subject Headings) terms. Example of the search strategy for PubMed (search strategies for all databases attached as supplementary file) is as follows:

((“uncontrolled hypertension”[Title/Abstract]) OR (“Hypertension control”) OR (“blood pressure control”) OR (“management of hypertension”) OR (“Treatment of Hypertension”)) AND ((“Diabetes Mellitus”) OR (“Type 2 diabetes mellitus”) OR (“Type 1 diabetes mellitus”) OR (“Diabetes”)) AND ( (Angola) OR (Benin) OR (Botswana) OR (Burkina Faso) OR (Burundi) OR (Cameroon) OR (Cape Verde) OR (Central African Republic) OR (Chad) OR (Comoros) OR (Congo) OR (Ivory Coast) OR (Democratic Republic of the Congo) OR (Djibouti) OR (Equatorial Guinea) OR (Eritrea) OR (Ethiopia) OR (Gabon) OR (Gambia) OR (Ghana) OR (Guinea) OR (Guinea-Bissau) OR (Kenya) OR (Lesotho) OR (Liberia) OR (Madagascar) OR (Malawi) OR (Mali) OR (Mauritania) OR (Mauritius) OR (Mayotte) OR (Mozambique) OR (Namibia) OR (Nigeria) OR (Reunion) OR (Rwanda) OR (Saint Helena) OR (Sao tome) OR (Senegal) OR (Seychelles) OR (Sierra Leone) OR (Somalia) OR (South Africa) OR (South Sudan) OR (Swaziland) OR (Togo) OR (Uganda) OR (Tanzania)OR (Zambia) OR (Zimbabwe)).

### Study selection

All studies retrieved using different electronic databases were exported into EndNote version X7. After excluding duplicated articles, titles of all articles were screened, and abstracts and their full texts were independently reviewed by two authors (YA and SAT). Disagreement between reviewers was resolved by further discussion and other reviewers (DAA and MG).

### Data extraction and management

Findings on the prevalence and associated factors of suboptimal blood pressure control among diabetic patients from each study were summarized by two authors (YA & YY) using the data extraction format which was prepared with the assistance of the Joanna Briggs Institute (JBI) data extraction tool for prevalence studies, and the extracted data were compared between the two authors (YA and YY). Discrepancies were resolved by consensus after discussion. For each study, the name of the first author, year of publication, study design, sample size, blood pressure cut point used (to define suboptimal blood pressure control), the prevalence of suboptimal blood pressure control, or the number of cases with suboptimal blood pressure control, and associated factor (sex, BMI, adherence) estimates (odds ratio or the cases in each cell) with their standard error were extracted.

### Risk of bias and quality assessment

The quality of included studies was assessed using the validated modified version of a quality assessment tool for prevalence studies [34]. Two reviewers (BD and GAT) independently assessed the quality of the included studies, and the discrepancy in quality appraisal between the two authors was synchronized by the third reviewer (SAT). The quality assessment tool has 9 risk of bias items which have a maximum score of “9” and a minimum score of “0.” The ranking of risk of bias is labeled as low risk (0–3), moderate risk (4–6), and high risk (7–9) [34].

### Data synthesis and analysis

The extracted data in Microsoft Excel were exported into STATA version 16.0 software for further analysis. The pooled estimate of the prevalence of suboptimal blood pressure control and its associated factors was determined by the random-effects model using DerSimonian-Laird weight [35]. Statistical heterogeneity was checked by Cochrane *Q*-test and *I*^2^ statistics [36]. To minimize the variance of point estimates between primary studies, subgroup analysis was carried out by BP cut point used to define suboptimal BP control, year of publication, income level, type of DM, and sample size. Besides, sensitivity analysis was also conducted to determine the effect of single studies on the pooled estimate. Moreover, univariable meta-regression was conducted by publication year, mean age of the respondent from primary studies, sample size, and income level using a random-effects model.

### Publication bias

Publication bias (small study effect) was checked using funnel plot and statistically by Egger’s test [37]. Odds ratio (OR) and 95% confidence interval were used to identify factors associated with suboptimal blood pressure control in people living with DM.

## Results

### Description of included studies

**Fig. 1:**
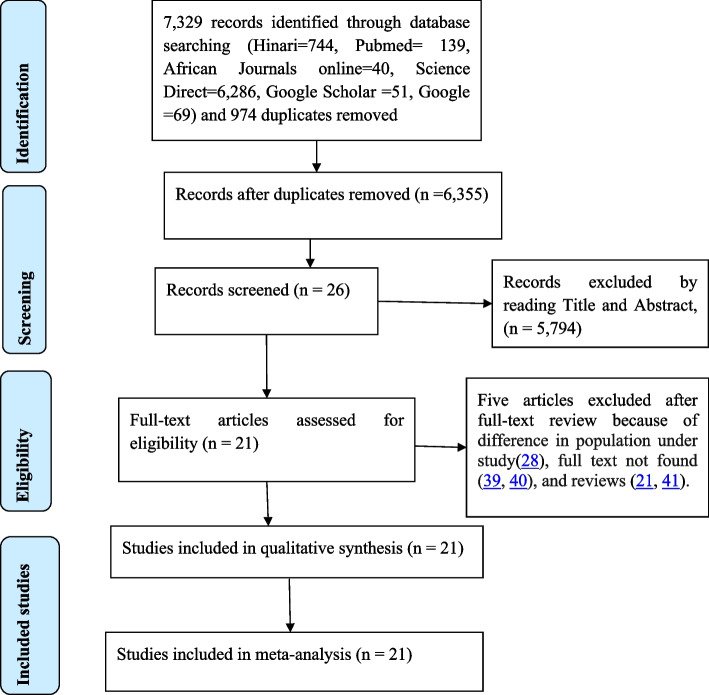
PRISMA flow diagram for a systematic review and meta-analysis of suboptimal blood pressure control among people living with diabetes in sub-Saharan African countries

We retrieved 7329 records of journal articles in the electronic database search, and 974 duplicates were removed. After a scrupulous review of the titles and abstracts, we excluded 5794 articles. Five articles were excluded in the full-text review because of differences in the population under study [27], full text not found [38, 39], and reviews [20, 40]. For further review, we used full-text copies of 21 records with an overall sample size of 6308 (Fig. 1).

Of the 21 studies, 5 were from South Africa [41–45], 5 from Ethiopia [10, 46–49], 3 from Kenya [50–52], 2 from Tanzania [53, 54], 2 from Nigeria [55, 56], and 1 from Cameron [24], Uganda [57], Botswana [58], and Ghana [59]. All studies were institution-based cross-sectional studies. To define suboptimal blood pressure control, BP cut point of ≥ 140/90 was used by 11 studies [10, 42, 45–47, 49, 52, 56, 58, 60, 61], ≥ 140/80 by 3 studies [44, 57], and ≥ 130/80 by 7 studies [24, 43, 51, 53–55] (Table 1).

**Table 1:** Characteristics of the included studies and prevalence of suboptimal blood pressure in individual studies

S. no	Author	Publication year	Country	Sample size	BP cut point used	Prevalence of suboptimal BP control	Quality score
1	N. S. Levitt et al. [42]	1997	South Africa	300	140/90	61.5	2 (low risk)
2	E. O. Okoro et al. [56]	2004	Nigeria	115	140/90	89	3 (low risk)
3	Simeon Pierre et al. [24]	2007	Cameroon	210	130/80	89.8	2 (low risk)
4	A. M. Klisiewicz et al. [41]	2009	South Africa	150	130/80	82	2 (low risk)
5	Anakwue R.C. et al. [55]	2012	Nigeria	420	130/80	88	0 (low risk)
6	Mwita J.C. et al. [53]	2012	Tanzania	150	130/80	66	2 (low risk)
7	Y. Pinchevsky et al. [43]	2013	South Africa	666	130/80	54.2	0 (low risk)
8	B. W. NDEGE et al. [51]	2014	Kenya	218	130/80	79	2 (low risk)
9	Kibirige et al. [57]	2014	Uganda	250	140/80	44	2 (low risk)
10	Hailu Abera et al. [46]	2016	Ethiopia	382	140/90	85	2 (low risk)
11	Oladele Vincent A. et al. [45]	2016	South Africa	265	140/90	75.5	3 (low risk)
12	Yacob Pinchevsky et al. [44]	2016	South Africa	261	140/80	58	3 (low risk)
13	Hailu A. et al. [48]	2017	Ethiopia	484	140/80	63.6	1 (low risk)
14	Mercy W. Kimando1 [61]	2017	Kenya	385	140/90	76.6	2 (low risk)
15	Semvua B1. Kilonzo et al. [54]	2017	Tanzania	295	130/80	84.5	2 (low risk)
16	Sintayehu Muleta et al. [10]	2017	Ethiopia	131	140/90	56.5	3 (low risk)
17	James Osei-Yeboah et al. [60]	2018	Ghana	150	140/90	41.33	3 (low risk)
18	Tariku Shimels et al. [49]	2018	Ethiopia	361	140/90	80.6	2 (low risk)
19	Emmanuel M. Mwengi et al. [52]	2019	Kenya	237	140/90	69.2	2 (low risk)
20	Julius Chacha Mwita et al. [58]	2019	Botswana	500	140/90	45.8	1 (low risk)
21	Akalu et al. [47]	2020	Ethiopia	378	140/90	74.4	2 (low risk)

### The pooled estimate of suboptimal blood pressure control among people living with DM in sub-Saharan Africa

**Fig. 2:**
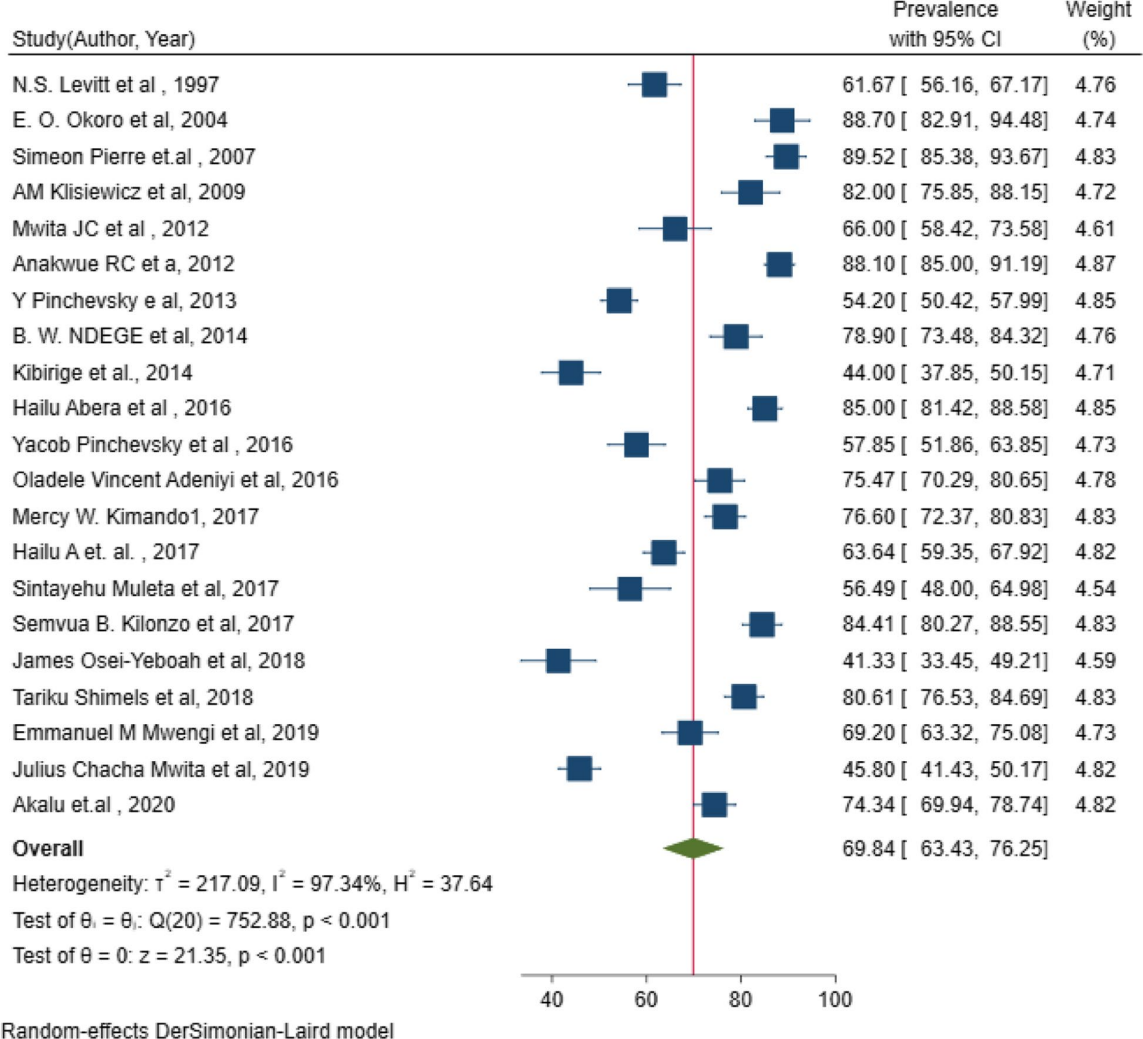
Forest plot for meta-analysis of suboptimal blood pressure control among DM patients in sub-Saharan African countries (*N* = 21)

**Fig. 3:**
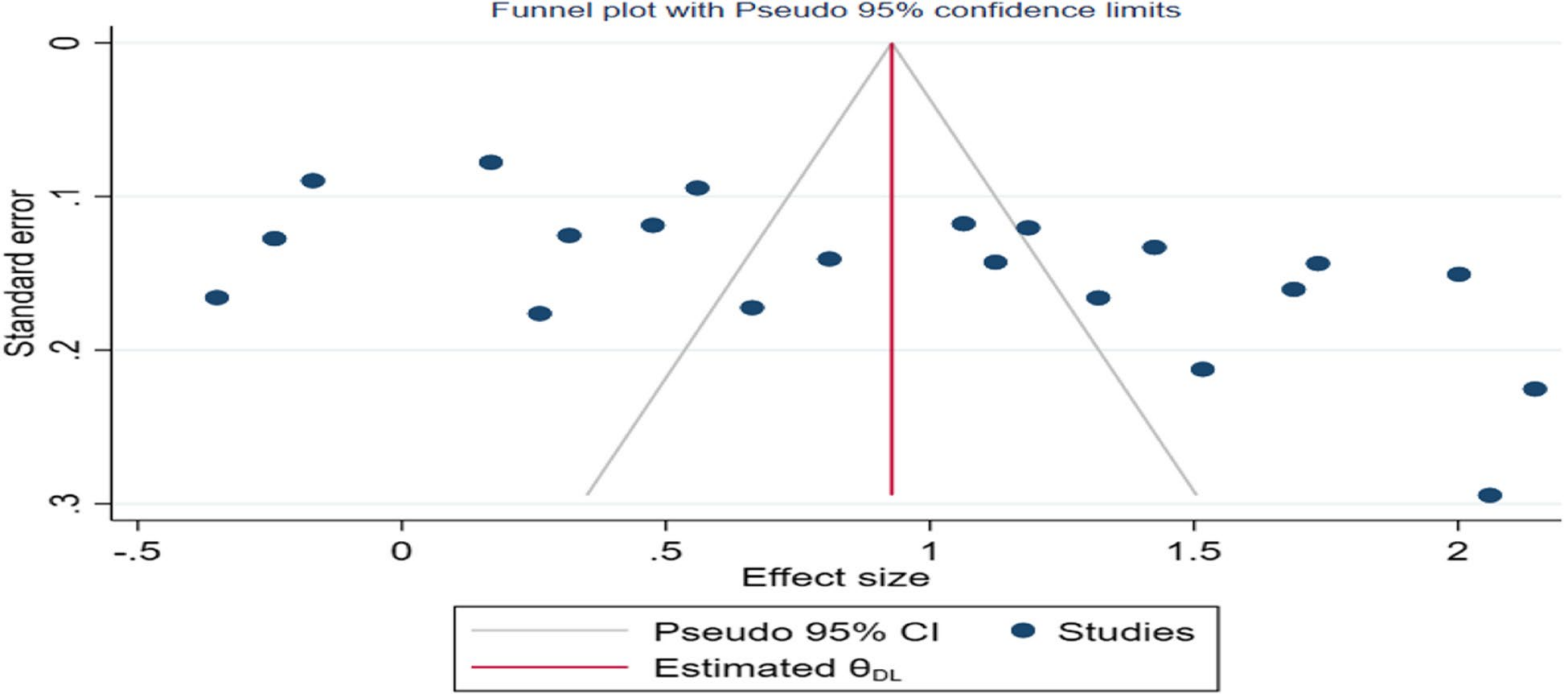
Funnel plot testing publication bias (random, *N* = 21)

The pooled prevalence of suboptimal blood pressure control in 21 studies in sub-Saharan Africa was 69.8% (95% *CI*: 63.43, 76.25%), and considerable heterogeneity was observed among studies (I^2^ = 97.3%, *p* < 0.001) (Fig. 2). The funnel plot shows the symmetric distribution, and the Egger’s test was not significant (estimated bias coefficient = 10.5 with a standard error of 3.4 and *p* = 0.180), indicating no publication bias (Fig. 3).

### Handling heterogeneity

**Fig. 4:**
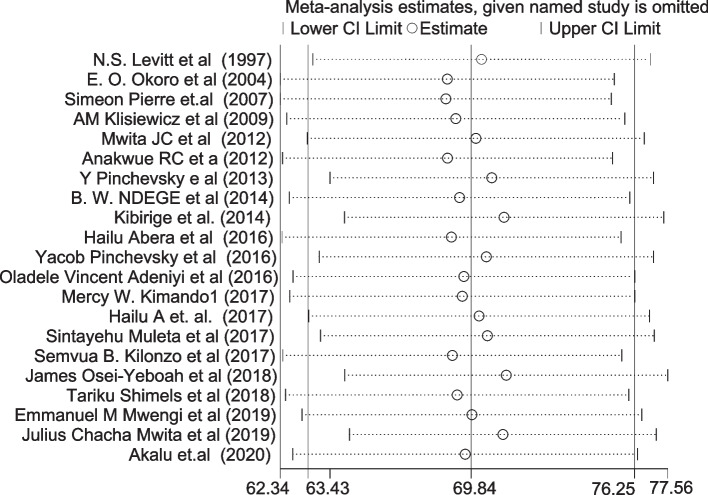
Sensitivity analysis between studies included in the meta-analysis

**Fig. 5:**
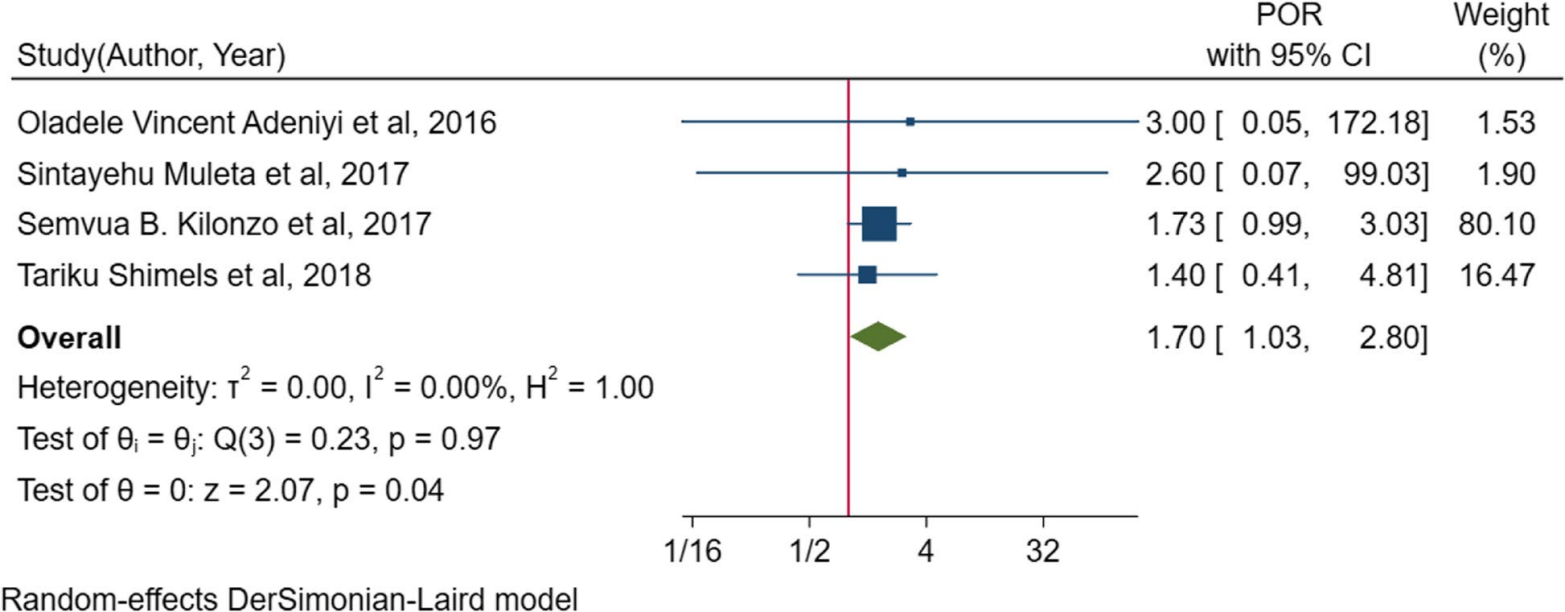
Forest plot for the association between poor adherence to antihypertensive drugs and suboptimal blood pressure control among diabetic peoples in sub-Saharan countries

The extent of heterogeneity among the included studies was high in the random-effects model pooled estimate. To handle this, sensitivity, subgroup, and meta-regression analyses were performed. In the sensitivity analysis, no influential study was found (Fig. 4). We further did and reported estimates from a sub-group analysis considering other possible sources of variations including the cut point used to diagnose suboptimal BP control, type of DM, sample size, income, and year of publication. However, heterogeneity was not handled. A pooled prevalence from sub-group analysis showed studies that used BP cut point of 130/80 mmHg systolic and diastolic blood pressure, respectively, had the highest prevalence of suboptimal BP control (77.7%) (Supplementary figure (S. Figure 1)), and the least pooled prevalence (55.3%) was observed in the subgroup of studies, which used 140/80 mmHg as a BP cut point. The pooled prevalence of suboptimal BP control was higher (71%) in studies whose study population was both (types 1 and/or 2) types of DM than studies with a study population of type 2 DM only (68.8%) (S. Figure 2). A higher pooled prevalence of suboptimal blood pressure control (70.0%) was found in studies with samples greater than or equal to 296 than the counterparts (S. Figure 3). Studies published from 2005 to 2012 had the highest pooled prevalence of suboptimal blood pressure control (82.0%) (S. Figure 4). Subgroup analysis by income was also done, and the highest pooled prevalence was found in lower-middle-income SSA countries (Ghana, Nigeria, Cameron) (77.3%) followed by low-income SSA countries (Ethiopia, Kenya, Uganda, Tanzania) (71.1%) (S. Figure 5) (Table 2).

**Table 2:** Subgroup analysis of the pooled prevalence of suboptimal BP control among the diabetic population in sub-Saharan African countries, 2020 (*n* = 21)

Subgroup analysis		Included studies	Sample size	Prevalence (95% *CI*)	Heterogeneity (*I*^2^, *p*-value)
BP cut point used	130/80	7	2,525	77.7% (66.92, 88.39)	97.5%, < 0.001
	140/80	3	995	55.3% (43.92, 66.71)	92.4%, < 0.001
	140/90	11	2788	68.8% (60.21, 77.47)	97.3%, < 0.001
Type of DM	Type 2 DM	11	3,754	68.8% (59.49, 78.02)	97.3%, < 0.001
	Both types 1 and 2	10	2554	71% (61.83, 80.24)	96.9%, < 0.001
Sample size	≤ 296	12	2432	69.7% (60.74, 70.61)	96.6%, < 0.001
	> 296	9	3876	70.0% (60.19, 79.88)	98.1%, < 0.001
Publication year	1997–2004	2	415	75.2% (48.68, 101.7)	97.7%, < 0.001
	2005–2012	4	930	82.3% (74.04, 90.01)	90.8%, < 0.001
	2013–2020	15	4,963	66.0% (58.59, 73.44)	97.2%, < 0.001
Income level	Low income	11	3271	71.1% (64.35, 77.82)	95.3%, < 0.001
	Lower middle income	4	895	77.3% (61.81, 92.77)	97.7%, < 0.001
	Upper middle income	6	2142	62.7 (52.15, 73.32)	96.3%, < 0.001

### Meta-regression

To handle heterogeneity, we further fitted meta-regression on the aggregated study level variables using the random-effects model. The univariable meta-regression analysis revealed that mean age, publication year, sample size, and income level were not significantly associated with suboptimal blood pressure control (Table 3).

**Table 3:** Univariable meta-regression analysis results for the prevalence of suboptimal blood pressure control among diabetics in sub-Saharan Africa

Study level variables	Adjusted *R*^2^	Standard error	Coefficients (95% *CI*)
Mean age	0.00	0.94	0.35 (− 1.49–2.19)
Publication year	0.00	0.60	− 0.62 (− 1.80, 0.54)
Sample size	4.40	0.02	− 0.02 (− 0.06, 0.03)
Income level	17.72	3.38	− 4.56 (− 11.18, 2.06)

### Factors associated with suboptimal blood pressure control among people living with diabetes mellitus in sub-Sahara Africa

**Fig. 6:**
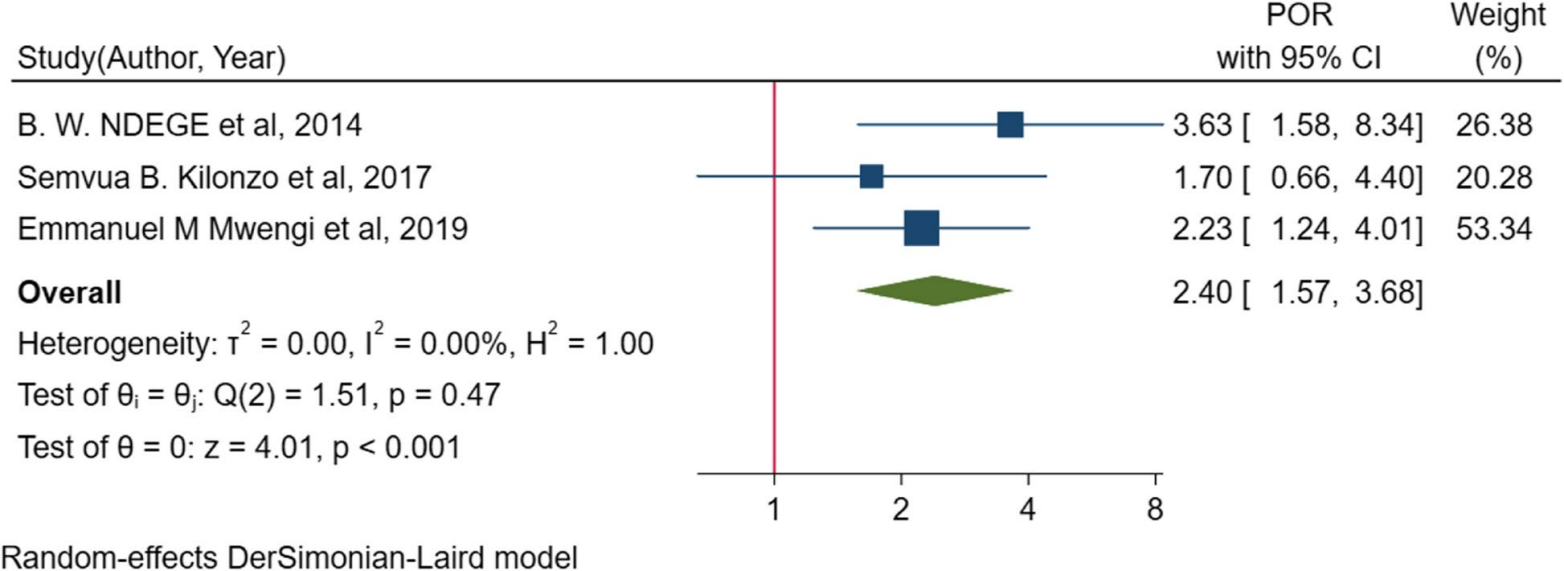
Forest plot for the association between BMI and suboptimal blood pressure control among diabetic peoples in sub-Saharan countries

**Fig. 7:**
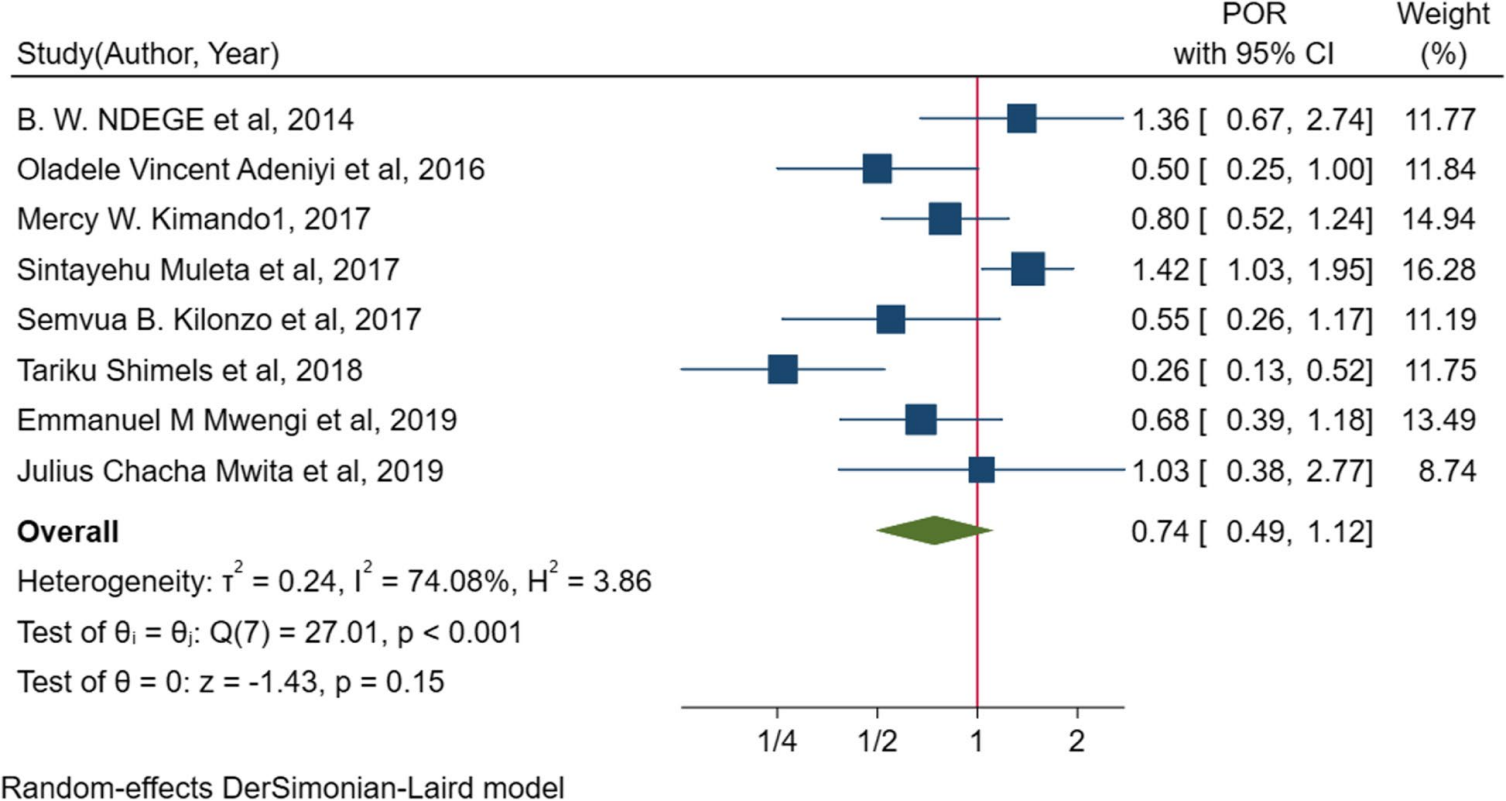
Forest plot for the association between sex (being female) and suboptimal blood pressure control among diabetic peoples in sub-Saharan countries

In the random effect model of meta-analysis of identified associated factors, the pooled effect of four studies [10, 45, 49, 54] showed that poor adherence to antihypertensive treatments (*POR* = 1.7; 95% *CI*: 1.03, 2.80, *I*^2^ = 0.0%, *p* = 0.531) was significantly associated with suboptimal blood pressure control among people living with diabetes mellitus (Fig. 5). The pooled effect of other three studies [51, 52, 54] also showed that body mass index (BMI) of ≥ 25 kg/m^2^ (*POR* = 2.4, 95% *CI*: 1.57, 3.68), *I*^2^ = 0.00%, *p* = 0.47) was significantly associated with suboptimal blood pressure control (Fig. 6). On the other hand, the pooled effect of eight studies [10, 45, 49, 51, 52, 54, 58, 61] showed that sex (*POR* = 0.7, 95% *CI*, 0.49–1.12, *I*^2^ = 74.1%, *p* = 0.001) was not significantly associated with suboptimal blood pressure control (Fig. 7). The likelihood of suboptimal blood pressure control among diabetic people with poor adherence to antihypertensive treatment was 1.7 times higher than their counterparts. Body mass index of ≥ 25 kg/m^2^ was associated with 2.4 times more odds of having suboptimal blood pressure control (Table 4).

**Table 4:** Summary of the pooled effects of factors associated with suboptimal blood pressure control among diabetics in sub-Saharan countries

Variables		OR (95% *CI*)	Heterogeneity (*I*^2^, *p*-value)	Egger’s *p*-value	Total studies	Sample size
Sex	Male	1				
	Female	0.7 (0.49–1.12)	74.1%, < 0.001	0.16	9	2,619
Poor adherence to antihypertensive treatments	Yes	1.7 (1.03, 2.80)*	0.0%, < 0.53	0.42	4	1,052
	No	1				
BMI (kg/m^2^)	< 25	1				
	≥ 25 kg/m^2^	2.4 (1.57, 3.68)*	0.0%, < 0.47	0.632	3	750

^*^Significant at *p* < 0.05

## Discussion

In people living with diabetes, the risk of developing CVD and life-threatening complications is determined by the degree of control of hypertension. However, hypertension is often inadequately controlled, and the cardiovascular complications in SSA diabetic individuals are attributed to suboptimal blood pressure control [26, 62]. Nevertheless, there is a paucity of data on the pooled prevalence and associated factors of suboptimal blood pressure control. Hence, this systematic review and meta-analysis estimated the pooled prevalence and associated factors of suboptimal blood pressure control among peoples living with diabetes mellitus in sub-Saharan Africa.

The pooled prevalence of suboptimal blood control in diabetes patients among 21 studies in SSA was 69.8% (95% *CI*: 63.43, 76.25%), which is in line with the study among diabetes patients in 11 sub-Saharan African countries (74.5%) [63]. However, due to the difference in blood pressure cut point used to diagnose suboptimal BP control by these studies, subgroup analysis was done by BP cut point. Accordingly, the pooled prevalence of suboptimal blood control among diabetes patients in seven SSA studies that use 130/80 mmHg as the cut point was 77.7% (95% *CI*: 66.92, 88.39) which is in line with a LEADER trial study among type 2 diabetes patients in 32 countries (74%) [64], a large population based study among Chinese, Malay, and Indian adults with diabetes and hypertension (87.2%) [65], a study in Brazil (67%) [66], and a systematic review and meta-analysis of 44 studies (88%) which use the same BP cut point to diagnose suboptimal blood pressure control [67]. The high prevalence of suboptimal BP control in people with diabetes might be due to different factors, such as giving more emphasis on glucose control and underemphasis on treatment for associated risk factors and morbidities such as hypertension [67]. In addition, inadequate access to follow-up care and prescription medications, inappropriate or ineffective treatments, poor adherence to prescription medication and lifestyle modifications, or a combination of these factors may be responsible [68, 69]. Low compliance is the main reason for suboptimal control of blood pressure in SSA [70]. This high prevalence of suboptimal blood pressure control among diabetes suggests an urgent need for initiatives to improve and control hypertension.

However, the prevalence of suboptimal BP control in this review is higher than the finding of a review of 16 studies (64.8%) [71], a study in the Netherland (62%) [72], and the result of the Hispanic community health study (51.3%) [73]. This inconsistency could be explained by the fact that patients in SSA countries have low socioeconomic status and suboptimal wealth index that are attributed to the weak health systems resulting in poor access to medications and a healthier lifestyle [74, 75]. They have low access to quality healthcare services and are more likely to have a low healthier lifestyle and to be non-adherent to their medications due to barriers in accessing medical care, unaffordable healthcare costs, lack of transport money to visit the hospital, and other reasons, leading to suboptimal blood pressure control [76, 77]. A great proportion of populations in SSA countries have no access to more than one blood pressure-lowering drug, and when they are available, they are not affordable [78]. Furthermore, poor dietary quality and practice such as high-saturated and trans-fatty acid intake, low fruit and vegetable consumption, and physical inactivity are increasingly becoming prevalent in low- and middle-income countries (LMICs) [75]. The mean salt intake in most of the LMICs is also beyond the recommended maximum intake. These all factors might contribute to the higher prevalence of suboptimal BP control in SSA.

On the contrary, the prevalence of suboptimal BP control among peoples living with diabetes in this review is lower than the prevalence of suboptimal blood pressure control in Spain (90.2%) [79]. This variation may be due to differences in the following: the proportion of overweight and obesity, the magnitude of comorbidity, age of study participants, and patient, physician, and sociocultural factors, which are hypothesized to have an impact on BP control [80].

On the other nine studies of this review which used a BP cut point of 140/90, the pooled prevalence of suboptimal blood pressure control was 70.0% (95% *CI*: 61.9%, 78.08). A similar study that uses the same BP cut point to diagnose suboptimal blood pressure control in Saudi Arabia among 1178 diabetic reported a consistent finding (71.8%) [81]. In this systematic review and meta-analysis, the lowest pooled prevalence of suboptimal blood pressure control (55.3%) was found in three studies that use BP cut point of 140/80 which is in line with the studies in South Africa (57.4%) [82] and Tanzania (57.8%) [83] that use the same BP cut point. This finding is lower than any of the above prevalence reports, which is clearly due to the BP cut point used to diagnose suboptimal BP control.

In this review, diabetes patients with poor adherence to antihypertensive medications were more likely to have suboptimal blood pressure control. This finding is in agreement with existing literature showing a higher prevalence of suboptimal blood pressure control among patients with poor adherence [84]. This is due to the fact that adherence to antihypertensive therapies is a primary determinant of treatment success, and poor medication adherence is the primary cause for suboptimal control of BP [85]. Poor adherence attenuates the effectiveness of antihypertensive drugs. Moreover, it is noticeable that poorly adherent patients are less likely to undertake a healthier lifestyle, contributing to suboptimal BP control [86]. Therefore, this review indicates the need for counseling and encouraging patients to adhere, as adherence to antihypertensive medications is a key to achieving an optimal BP.

In the current review, a BMI of 25 kg/m^2^ or greater was associated with suboptimal blood pressure control. Consistently, other studies reported that being obese is associated with suboptimal blood pressure control [81, 87, 88]. In obesity, there is an increased production of leptin, a polypeptide produced from adipocytes that stimulates sympathetic activity leading to renal water retention, increased heart rate, and peripheral vascular resistance, and finally increased blood pressure beyond the target [85]. Insulin and leptin-induced activation of the sympathetic nervous system is also associated with tubular sodium reabsorption and volume expansion and consequently increased blood pressure [89]. Moreover, an increase in BMI results in elevation of plasma aldosterone [90], thereby causing suboptimal blood pressure control.

Findings from the current review might be helpful for clinicians, programmers, and policymakers to design a strategy and take prompt interventions which would prevent complications and death due to uncontrolled hypertension in people living with diabetes and hypertension comorbidity.

### Limitations

Though sensitivity, subgroup, and meta-regression analyses were conducted to minimize the effect of heterogeneity, the extent of heterogeneity among the included studies was high which might be due to the difference in the study area, methodology, study period, blood pressure cut point used to diagnose suboptimal blood pressure control, and other unexplained variations. Hence, clinicians and policymakers should consider these during the interpretations of results.

### Future directions

It would be better if future efforts focused on identifying further causes of high prevalence of suboptimal blood pressure control among diabetes patients in SSA on a larger sample size. Moreover, clinicians and policymakers should concentrate on controlling modifiable risk factors of suboptimal blood pressure control.

## Conclusion

The pooled prevalence of suboptimal blood pressure control among people with diabetes was high. This high prevalence of suboptimal BP in hypertensive diabetic people highlights an urgent need for initiatives to improve and control hypertension. BMI of 25 kg/m^2^ or greater and poor adherence to antihypertensive treatments were significantly associated with suboptimal blood pressure control. Preventive measures should concentrate on overweight patients and patients with poor adherence to antihypertensive medications. 

## Supplementary Information

**Additional file 1: S. Figure 1** Forest plot for meta-analysis of suboptimal blood pressure control sub-analyzed by blood pressure cut point used for diagnosis of suboptimal blood pressure control among diabetes patients in sub-Saharan Africa countries (*N* = 21, random effect model). **S. Figure 2** Forest plot for meta-analysis of suboptimal blood pressure control among diabetes patients in sub-Saharan Africa countries sub-analyzed by Type of DM (*N* = 21, random effect model). **S. Figure 3** Forest plot for meta-analysis of suboptimal blood pressure control among diabetes patients in sub-Saharan Africa countries sub-analyzed by sample size (*N* = 21, random effect model). **S. Figure 4** Forest plot for meta-analysis of suboptimal blood pressure control among diabetes patients in sub-Saharan Africa countries sub-analyzed by publication year (*N* = 21, random effect model).** S. Figure 5** Forest plot for meta-analysis of suboptimal blood pressure among diabetes patients in sub-Saharan Africa countries sub-analyzed by income (*N* = 21, random effect model).

**Additional file 2:** Search strategies of data bases used for the systematic review and meta-analysis on prevalence and associated factors of suboptimal blood pressure control among diabetes mellitus patients in sub-Saharan Africa.

## Data Availability

All the materials and data are presented within the manuscript.

## Abbreviations

BP: Blood pressure
BMI: Body mass index
DBP: Diastolic blood pressure
DM: Diabetes mellitus
LMICs: Low- and middle-income countries
SBP: Systolic blood pressure
SSA: Sub-Saharan African countries
T2DM: Type 2 diabetes mellitus
