# Supplementary material for: Suboptimal blood pressure control and its associated factors among people living with diabetes mellitus in sub-Saharan Africa: a systematic review and meta-analysis
Source: Syst Rev. 2022 Oct 15;11:220. doi: 10.1186/s13643-022-02090-4 (PMC9569048; doi:10.1186/s13643-022-02090-4)
Supplement: Supplementary file 1 — Additional file 1: S. Figure 1 Forest plot for meta-analysis of suboptimal blood pressure control sub-analyzed by blood pressure cut point used for diagnosis of suboptimal blood pressure control among diabetes patients in sub-Saharan Africa countries (N = 21, random effect model). S. Figure 2 Forest plot for meta-analysis of suboptimal blood pressure control among diabetes patients in sub-Saharan Africa countries sub-analyzed by Type of DM (N = 21, random effect model). S. Figure 3 Forest plot for meta-analysis of suboptimal blood pressure control among diabetes patients in sub-Saharan Africa countries sub-analyzed by sample size (N = 21, random effect model). S. Figure 4 Forest plot for meta-analysis of suboptimal blood pressure control among diabetes patients in sub-Saharan Africa countries sub-analyzed by publication year (N = 21, random effect model). S. Figure 5 Forest plot for meta-analysis of suboptimal blood pressure among diabetes patients in sub-Saharan Africa countries sub-analyzed by income (N = 21, random effect model). [file 13643_2022_2090_MOESM1_ESM.docx]

**Supplementary figures**

**
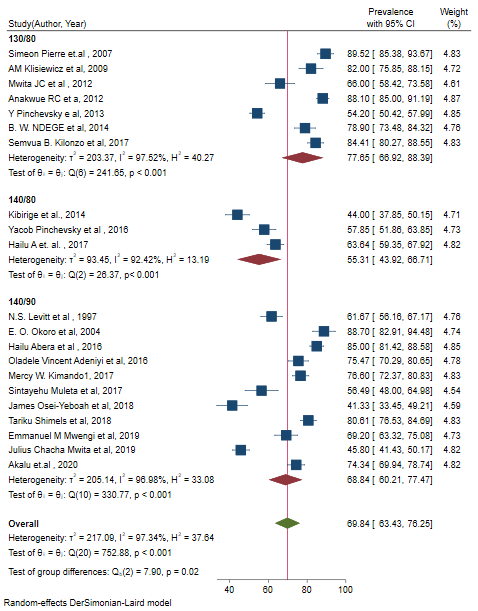
**

**S.Figure 1** Forest plot for meta-analysis of suboptimal blood pressure control sub-analyzed by blood pressure cut point used for diagnosis of suboptimal blood pressure control among diabetes patients in sub-Saharan Africa countries (N = 21, random effect model)


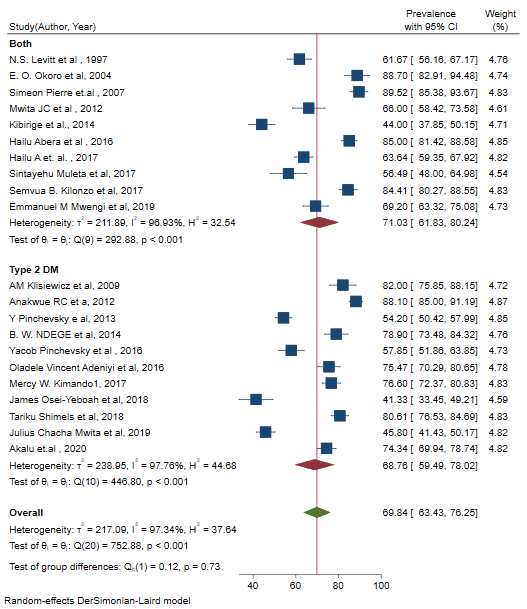


**S. Figure 2** Forest plot for meta-analysis of suboptimal blood pressure control among diabetes patients in sub-Saharan Africa countries sub-analyzed by Type of DM (N = 21, random effect model)


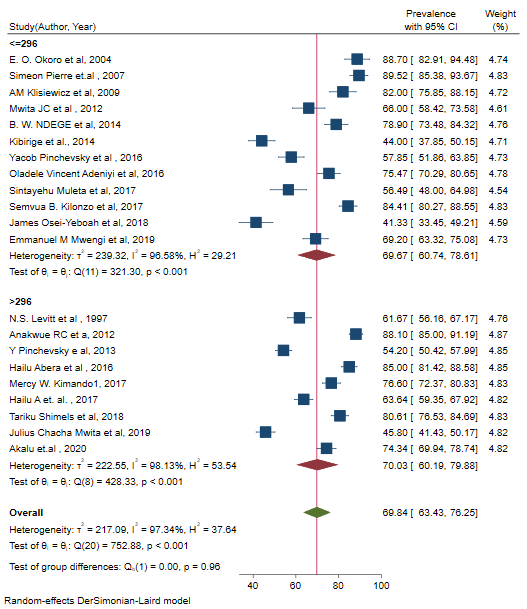


**S.Figure 3** Forest plot for meta-analysis of suboptimal blood pressure control among diabetes patients in sub-Saharan Africa countries sub-analyzed by sample size (N = 21, random effect model)


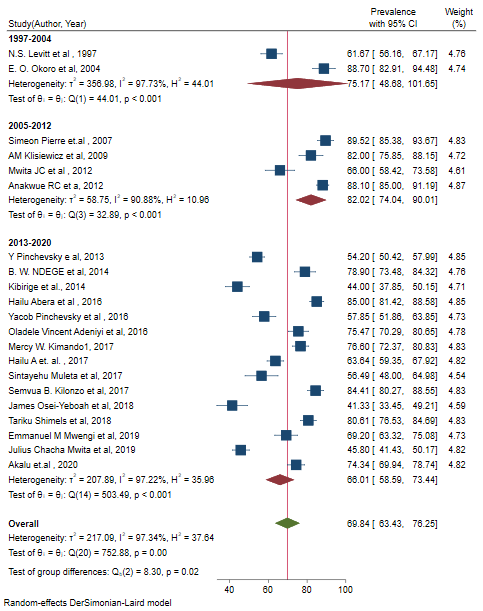


**S. Figure 4** Forest plot for meta-analysis of suboptimal blood pressure control among diabetes patients in sub-Saharan Africa countries sub-analyzed by publication year (N = 21, random effect model)

**
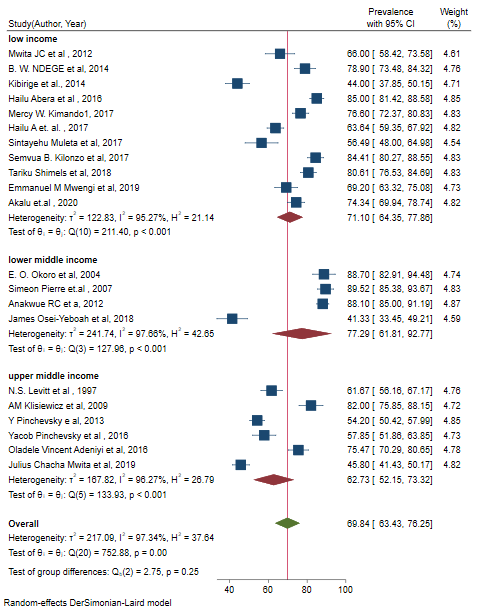
**

**S.Figure 5** Forest plot for meta-analysis of suboptimal blood pressure among diabetes patients in sub-Saharan Africa countries sub-analyzed by income (N = 21, random effect model)
