# Supplementary material for: Suboptimal blood pressure control and its associated factors among people living with diabetes mellitus in sub-Saharan Africa: a systematic review and meta-analysis
Source: Syst Rev. 2022 Oct 15;11:220. doi: 10.1186/s13643-022-02090-4 (PMC9569048; doi:10.1186/s13643-022-02090-4)
Supplement: Supplementary file 2 — Additional file 2: Search strategies of data bases used for the systematic review and meta-analysis on prevalence and associated factors of suboptimal blood pressure control among diabetes mellitus patients in sub-Saharan Africa. [file 13643_2022_2090_MOESM2_ESM.docx]

**Search strategies of data bases used for the systematic review and meta-analysis on prevalence and associated factors of suboptimal blood pressure control among diabetes mellitus patients in sub-Saharan Africa.**

Hinari, PubMed, African Journals online, Science District, Google scholar, and direct Google search were used to access relevant studies for this review.

1. **Advanced Hinari search**

- A total of =744 articles has been retrieved using the following search strategies that in corporate name of all sub-Saharan Countries

((TitleCombined:(uncontrolled hypertension)) OR (TitleCombined:(Hypertension control)) OR (TitleCombined:(blood pressure control))) OR (TitleCombined:(management of hypertension)) OR (TitleCombined:(Treatment of Hypertension))) AND ((TitleCombined:(Diabetes Mellitus)) OR (TitleCombined:(Type 2 diabetes mellitus)) OR (TitleCombined:(Type 1 diabetes mellitus)) OR (TitleCombined:(Diabetes))) AND ((TitleCombined:(Angola)) OR (TitleCombined:(Benin)) OR (TitleCombined:(Botswana)) OR (TitleCombined:(Burkina faso)) OR (TitleCombined:(Burndi)) OR (TitleCombined:(Cameroon)) OR (TitleCombined:(Cape Verde)) OR (TitleCombined:(Central Africa Republic)) OR (TitleCombined:(Chad)) OR (TitleCombined:(Comoros)) OR (TitleCombined:(Congo))) =0

((Abstract:(uncontrolled hypertension)) OR (Abstract:(Hypertension control)) OR (Abstract:(blood pressure control))) OR (Abstract:(management of hypertension)) OR (Abstract:(Treatment of Hypertension))) AND ((Abstract:(Diabetes Mellitus)) OR (Abstract:(Type 2 diabetes mellitus)) OR (Abstract:(Type 1 diabetes mellitus)) OR (Abstract:(Diabetes))) AND ((Abstract:(Angola)) OR (Abstract:(Benin)) OR (Abstract:(Botswana)) OR (Abstract:(Burkina faso)) OR (Abstract:(Burndi)) OR (Abstract:(Cameroon)) OR (Abstract:(Cape Verde)) OR (Abstract:(Central Africa Republic)) OR (Abstract:(Chad)) OR (Abstract:(Comoros)) OR (Abstract:(Congo)))=**134 articles**

((TitleCombined: (uncontrolled hypertension)) OR (TitleCombined:(Hypertension control)) OR (TitleCombined:(blood pressure control)) OR (TitleCombined:(management of hypertension)) OR (TitleCombined:(Treatment of Hypertension))) AND ((TitleCombined :(Diabetes Mellitus)) OR (TitleCombined:(Type 2 diabetes mellitus)) OR (TitleCombined:(Type 1 diabetes mellitus))) AND ((TitleCombined:(Democratic Republic of the Congo)) OR (TitleCombined:(Djibouti)) OR (TitleCombined:(Equatorial Guinea)) OR (TitleCombined:(Eritrea)) OR (TitleCombined:(Ethiopia)) OR (TitleCombined:(Gabon)) OR (TitleCombined:(Gambia)) OR (TitleCombined:(Ghana)) OR (TitleCombined:(Guinea)) OR (TitleCombined:(Guinea-Bissau)) OR (TitleCombined:(Kenya)) OR (TitleCombined:(Lesetho)))=**1 article found**

((Abstract: (uncontrolled hypertension)) OR (Abstract:(Hypertension control)) OR (Abstract:(blood pressure control)) OR (Abstract:(management of hypertension)) OR (Abstract:(Treatment of Hypertension))) AND ((Abstract:(Diabetes Mellitus)) OR (Abstract:(Type 2 diabetes mellitus)) OR (Abstract:(Type 1 diabetes mellitus))) AND ((Abstract:(Democratic Republic of the Congo)) OR (Abstract:(Djibouti)) OR (Abstract:(Equatorial Guinea)) OR (Abstract:(Eritrea)) OR (Abstract:(Ethiopia)) OR (Abstract:(Gabon)) OR (Abstract:(Gambia)) OR (Abstract:(Ghana)) OR (Abstract:(Guinea)) OR (Abstract:(Guinea-Bissau)) OR (Abstract:(Kenya)) OR (Abstract:(Lesetho)))=**87 articles retrieved**

((TitleCombined:(uncontrolled hypertension)) OR (TitleCombined:(Hypertension control)) OR (TitleCombined:(blood pressure control)) OR (TitleCombined:(management of hypertension)) OR (TitleCombined:(Treatment of Hypertension))) AND ((TitleCombined:(Diabetes Mellitus)) OR (TitleCombined:(Type 2 diabetes mellitus)) OR (TitleCombined:(Type 1 diabetes mellitus)) OR (TitleCombined:(Diabetes))) AND ((TitleCombined:(Madagascar)) OR (TitleCombined:(Malawi)) OR (TitleCombined:(Mali)) OR (TitleCombined:(Mauritania)) OR (TitleCombined:(Mauritius)) OR (TitleCombined:(Mayotte)) OR (TitleCombined:(Mozambic)) OR (TitleCombined:(Namibia)) OR (TitleCombined:(Niger)) OR (TitleCombined:(Nigeria)) OR (TitleCombined:(Reunion)))=**5 articles retrieved**

((Abstract:(uncontrolled hypertension)) OR (Abstract:(Hypertension control)) OR (Abstract:(blood pressure control)) OR (Abstract:(management of hypertension)) OR (Abstract:(Treatment of Hypertension))) AND ((Abstract:(Diabetes Mellitus)) OR (Abstract:(Type 2 diabetes mellitus)) OR (Abstract:(Type 1 diabetes mellitus)) OR (Abstract:(Diabetes))) AND ((Abstract:(Madagascar)) OR (Abstract:(Malawi)) OR (Abstract:(Mali)) OR (Abstract:(Mauritania)) OR (Abstract:(Mauritius)) OR (Abstract:(Mayotte)) OR (Abstract:(Mozambique)) OR (Abstract:(Namibia)) OR (Abstract:(Niger)) OR (Abstract:(Nigeria)) OR (Abstract:(Reunion)))=**191 articles retrieved**

(TitleCombined:(blood pressure control)) OR (TitleCombined:(management of hypertension)) OR (TitleCombined:(Treatment of Hypertension))) AND ((TitleCombined:(Diabetes Mellitus)) OR (TitleCombined:(Type 2 diabetes mellitus)) OR (TitleCombined:(Type 1 diabetes mellitus)) OR (TitleCombined:(Diabetes))) AND ((TitleCombined:( Liberia)) OR (TitleCombined:( Rwanda)) OR (TitleCombined:( Sao tome)) OR (TitleCombined:( Senegal)) OR (TitleCombined:( Seychelles)) OR (TitleCombined:( Sierra Leone)) OR (TitleCombined:( Somalia)) OR (TitleCombined:( South Africa)) OR (TitleCombined:( South Sudan)) OR (TitleCombined:( Swaziland)) OR (TitleCombined:( Togo)))=**2 articles retrieved**

((Abstract:(uncontrolled hypertension)) OR (Abstract:(Hypertension control)) OR (Abstract:(blood pressure control)) OR (Abstract:(management of hypertension)) OR (Abstract:(Treatment of Hypertension))) AND ((Abstract:(Diabetes Mellitus)) OR (Abstract:(Type 2 diabetes mellitus)) OR (Abstract:(Type 1 diabetes mellitus)) OR (Abstract:(Diabetes))) AND ((Abstract:( Liberia)) OR (Abstract:( Rwanda)) OR (Abstract:( Sao tome)) OR (Abstract:( Senegal)) OR (Abstract:( Seychelles)) OR (Abstract:( Sierra Leone)) OR (Abstract:( Somalia)) OR (Abstract:( South Africa)) OR (Abstract:( South Sudan)) OR (Abstract:( Swaziland)) OR (Abstract:( Togo))) = **223 articles retrieved**

((TitleCombined:(uncontrolled hypertension)) OR (TitleCombined:(Hypertension control)) OR (TitleCombined:(blood pressure control)) OR (TitleCombined:(management of hypertension)) OR (TitleCombined:(treatment of hypertension))) AND ((TitleCombined:(Diabetes Mellitus)) OR (TitleCombined:(Type 2 diabetes mellitus)) OR (TitleCombined:(Type 1 diabetes mellitus)) OR(TitleCombined:(Diabetes)))AND((TitleCombined:(Uganda))OR(TitleCombined:(Tanzania)) OR (TitleCombined:(Zambia)) OR (TitleCombined:(Zimbabwe)))=**5 articles retrieved**

((Abstract:(uncontrolled hypertension)) OR (Abstract:(Hypertension control)) OR (Abstract:(blood pressure control)) OR (Abstract:(management of hypertension)) OR (Abstract:(treatment of hypertension))) AND ((Abstract:(Diabetes Mellitus)) OR (Abstract:(Type 2 diabetes mellitus)) OR (Abstract:(Type 1 diabetes mellitus)) OR (Abstract:(Diabetes))) AND ((Abstract:(Uganda)) OR (Abstract:(Tanzania)) OR (Abstract:(Zambia)) OR (Abstract:(Zimbabwe)))=**96 articles retrieved**

1. **PubMed**

- **Using advanced PubMed search we retrieved 139 articles**

(("uncontrolled hypertension"[Title/Abstract]) OR (“Hypertension control”) OR ("blood pressure control") OR ("management of hypertension") OR (“Treatment of Hypertension”)) AND ((“Diabetes Mellitus”) OR ("Type 2 diabetes mellitus") OR ("Type 1 diabetes mellitus") OR (“Diabetes”)) AND ( (Angola) OR (Benin) OR (Botswana) OR (Burkina Faso) OR (Burundi) OR (Cameroon) OR (Cape Verde) OR (Central African Republic) OR (Chad) OR (Comoros) OR (Congo) OR (Ivory Coast) OR (Democratic Republic of the Congo) OR (Djibouti) OR (Equatorial Guinea) OR (Eritrea) OR (Ethiopia) OR (Gabon) OR (Gambia) OR (Ghana) OR (Guinea) OR (Guinea-Bissau) OR (Kenya) OR (Lesotho) OR (Liberia) OR (Madagascar) OR (Malawi) OR (Mali) OR (Mauritania) OR (Mauritius) OR (Mayotte) OR (Mozambique) OR (Namibia) OR (Niger) OR (Nigeria) OR (Reunion) OR (Rwanda) OR (Saint Helena) OR (Sao tome) OR (Senegal) OR (Seychelles) OR (Sierra Leone) OR (Somalia) OR (South Africa) OR (South Sudan) OR (Swaziland) OR (Togo) OR (Uganda) OR (Tanzania)OR (Zambia) OR (Zimbabwe))

1. **African Journals Online**

**Forty articles (40)** were retrieved using African Journals Online by the following search strategy.

((uncontrolled hypertension) OR (Hypertension control) OR (blood pressure control) OR (management of hypertension) OR (Treatment of Hypertension)) AND ((Diabetes Mellitus) OR (Type 2 diabetes mellitus) OR (Type 1 diabetes mellitus) OR (Diabetes)) AND ( (Angola) OR (Benin) OR (Botswana) OR (Burkina Faso) OR (Burundi) OR (Cameroon) OR (Cape Verde) OR (Central African Republic) OR (Chad) OR (Comoros) OR (Congo) OR (Ivory Coast) OR (Democratic Republic of the Congo) OR (Djibouti) OR (Equatorial Guinea) OR (Eritrea) OR (Ethiopia) OR (Gabon) OR (Gambia) OR (Ghana) OR (Guinea) OR (Guinea-Bissau) OR (Kenya) OR (Lesotho) OR (Liberia) OR (Madagascar) OR (Malawi) OR (Mali) OR (Mauritania) OR (Mauritius) OR (Mayotte) OR (Mozambique) OR (Namibia) OR (Niger) OR (Nigeria) OR (Reunion) OR (Rwanda) OR (Saint Helena) OR (Sao tome) OR (Senegal) OR (Seychelles) OR (Sierra Leone) OR (Somalia) OR (South Africa) OR (South Sudan) OR (Swaziland) OR (Togo) OR (Uganda) OR (Tanzania)OR (Zambia) OR (Zimbabwe)) Sierra Leone) OR (Somalia) OR (South Africa) OR (South Sudan) OR (Swaziland) OR (Togo)

1. **Science Direct**

Six thousands two hundreds eighty six **(6,286)** were retrieved using Science Direct by the following search strategy with a custom range of 1997-2020.

“Uncontrolled hypertension or blood pressure control or management of hypertension AND Diabetes Mellitus”
